# Nonalcoholic fatty liver disease and type 2 diabetes: where do Diabetologists stand?

**DOI:** 10.1186/s40842-020-00097-1

**Published:** 2020-06-05

**Authors:** Shaheen Tomah, Naim Alkhouri, Osama Hamdy

**Affiliations:** 1 Research Division, Joslin Diabetes Center, 1 Joslin Place, Boston, MA 02215 USA; 2 Department of Medicine, Harvard Medical School, Boston, MA 02215 USA; 3 Texas Liver Institute, University of Texas (UT) Health, San Antonio, TX USA

**Keywords:** Type 2 diabetes, Nonalcoholic fatty liver disease, Nonalcoholic steatohepatitis, Pathophysiology, Awareness, Screening, Treatment

## Abstract

**Background:**

Nonalcoholic fatty liver disease (NAFLD) is the most common chronic liver disease worldwide. The increasing prevalence of NAFLD mirrors that of obesity and type 2 diabetes over the last two decades.

**Main:**

In a two-way pathophysiologic relationship, NAFLD increases the risk of developing type 2 diabetes, while the latter promotes the progression of simple fatty liver to a more advanced form called nonalcoholic steatohepatitis (NASH). NASH increases the risk of cirrhosis and hepatocellular carcinoma (HCC), which may require liver transplantation. With the absence of FDA-approved medications for NAFLD treatment, lifestyle intervention remains the only therapy. Lately, extensive research efforts have been aimed at modifying NASH fibrosis and developing noninvasive screening methods.

**Conclusion:**

We highlight the pathophysiologic relationships between NAFLD and type 2 diabetes, discuss disease recognition, models of care, and current and emerging therapies for NASH treatment.

## Background

**Fig. 1:**
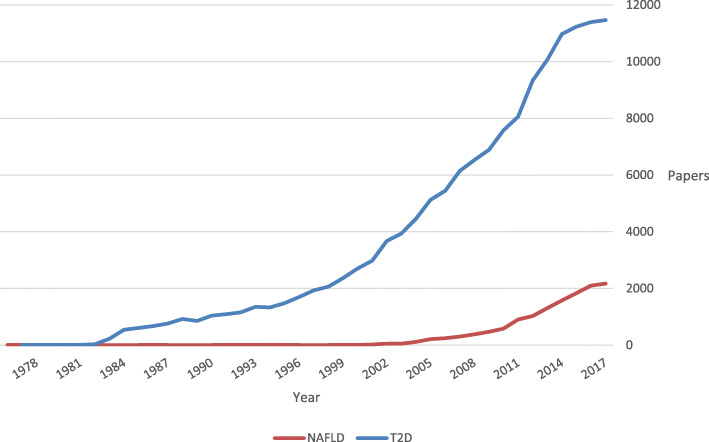
Nonalcoholic fatty liver disease and type 2 diabetes in publications over four decades. Based on data from Pubmed.gov literature search with the keywords: nonalcoholic fatty liver disease OR type 2 diabetes. Abbreviations: NAFLD, nonalcoholic fatty liver disease; T2D, type 2 diabetes

Nonalcoholic fatty liver disease (NAFLD) is an umbrella term that encompasses multiple progressive liver disorders, ranging from simple hepatic steatosis, often called nonalcoholic fatty liver (NAFL), to nonalcoholic steatohepatitis (NASH) which is marked by hepatocyte inflammation and ballooning. Around 35% of NASH cases progress to liver fibrosis [1] and potentially to end-stage liver disease or hepatocellular carcinoma (HCC) [2, 3]. The growing epidemic of NAFLD in western societies is estimated to affect around 20 to 30% of the overall population and 45 to 75% of patients with type 2 diabetes [4, 5]. Over the last two decades, the high prevalence rates of NAFLD have been paralleling the rapidly progressing epidemic of obesity and type 2 diabetes [6, 7]. In fact, we see NAFLD and type 2 diabetes at the intersection of similar risk factors, epidemiology, and pathophysiology [8–10]. In terms of precedence, the recognition of NAFLD as a major chronic disease is relatively new compared to type 2 diabetes [11]. This is also evident in medical literature over the past 40 years (Fig. 1). Currently, NAFLD is considered the most common chronic liver disease worldwide and a leading etiology of liver diseases among adults awaiting liver transplantation in the US [5, 12–14]. The co-existence of NAFLD and type 2 diabetes significantly increases the likelihood of developing NASH and cirrhosis compared to the presence of NAFLD without persistent hyperglycemia [10]. The involvement of NAFLD as an independent predictor of cardiovascular disease (CVD) events remains debatable [2, 15, 16]. Interestingly, the highest mortality in NAFLD is attributed not to end-stage liver disease, or risk of HCC, but to worse CVD risk profile [17] possibly driven by the comorbidity of type 2 diabetes and other established CVD risk factors [15]. Hence, increased risk of CVD in patients with type 2 diabetes and NAFLD may exert significant impact on their mortality. With recent advances in NAFLD diagnosis and many phase III trials for NASH-specific therapies, there is an essential evolving role for diabetologists in identifying patients with type 2 diabetes at high risk for NAFLD complications and initiating an integrated multidisciplinary plan of care to achieve the best possible results.

In this review, we highlight the pathophysiologic relationships between NAFLD and type 2 diabetes, discuss disease recognition, current therapeutic options, and summarize emerging novel therapies for NASH treatment.

## Main text

### Pathogenesis of NAFLD in relation to type 2 diabetes

**Fig. 2:**
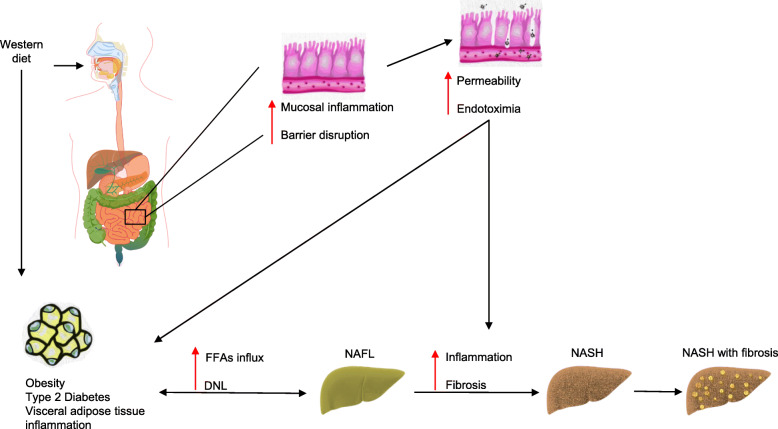
Key players in the development of NAFLD/NASH comprising mechanisms in the gut, adipose tissue and liver. Abbreviations: FFA, free fatty acid; NAFL, nonalcoholic fatty liver; DNL, de novo lipogenesis; NASH, nonalcoholic steatohepatitis

The pathogenesis of NAFLD has not been fully unraveled. Footprints of insulin resistance (IR) with associated subclinical inflammation is one of many that were recognized in the course of NAFLD. In this pro-inflammatory state, an increased influx of free fatty acids (FFAs) to the liver causes fatty infiltration in the hepatocytes, which induces liver damage via lipid peroxidation and mitochondrial dysfunction [18, 19]. Another important source of fatty acids and intrahepatic triglycerides in patients with NAFLD is de novo lipogenesis (DNL), even under fasting conditions, compared to obese patients without NAFLD [20]. Furthermore, obesity per se through adipose tissue inflammation and increased importation of FFAs to the liver has also been considered an important cause of hepatocellular injury [21]. Beyond obesity, chronic glucotoxicity aggravated by persistent hyperglycemia is a key phenomenon observed in the course of type 2 diabetes [22]. Glucotoxicity may promote the progression of NASH via glucose-induced IR, increased DNL, and hepatocellular dysfunction [23]. On the other hand, a recent animal study showed that dietary fructose, but not glucose, impaired fat metabolism via changes in mitochondrial morphology and function when added to a high-fat diet [24]. Many other factors are involved in the pathogenesis of NAFLD comprising mechanisms in the gut, adipose tissue, and liver (Fig. 2). These are often referred to in the literature as the gut-fat-liver axis [25]. Recent advances in multi-omics studies with gut microbiota profiling showed that increased metabolic endotoxemia due to high gut permeability is closely tied to the development and progression of NAFLD [26]. These consecutive or somewhat parallel mechanisms promote cell stress and apoptotic pathways. In NASH, Lipotoxicity-induced hepatocyte ballooning leads to downregulation of a key player in the apoptotic pathway, which is caspase 9, and along with reinforcement from a hedgehog autocrine survival signaling pathway produces an “undead hepatocyte” in which apoptosis has been initiated but fails to be executed driving a vicious pathway of inflammation (NASH) and fibrosis [27]. Among all the histologic features of NASH, fibrosis is the most important predictor of end-stage liver disease and increased mortality [28]. Obesity, metabolic endotoxemia, and IR are all hallmarks of the metabolic syndrome (MetS) and type 2 diabetes [29–31]. Now that NAFLD is considered by many as the hepatic manifestation of the MetS, the magnitude of the problem can be better appreciated [32, 33].

### Disease recognition

The diagnosis of NAFLD could be missed due to the lack of cost-effective, non-invasive diagnostic tools, and the absence of a clear consensus on the value of screening for NAFLD [2, 34–36]. A recent large-scale study reported a significant gap in diagnosing NAFLD based on primary-care records of almost 18 million adults from the UK, Italy, Spain, and the Netherlands [37]. This mandates a multidisciplinary approach aimed to identify patients at risk for developing the advanced form of the disease and accelerate the advancement of research to explore new targets for therapy and reliable serum-based biomarkers. Although it is well-established that patients with type 2 diabetes are at a substantially increased risk for NAFLD complications, patients with type 1 diabetes who are overweight or obese may not be immune. Currently, around 50% of patients with type 1 diabetes have weight problems; a condition frequently named double-diabetes [38, 39]. Despite these concerns, it is still debated whether to screen patients with diabetes for NAFLD or not [2, 35].

#### NASH as a complication of type 2 diabetes

The relationship between NAFLD and type 2 diabetes is bidirectional [6, 40]. Diabetes promotes the progression of NAFL to NASH and increases the risk of cirrhosis and HCC. On the other hand, NAFLD is associated with an increased risk of developing type 2 diabetes [8–10].

In 1980, Ludwig et al. coined the term “nonalcoholic steatohepatitis” after reporting a case-series of 20 patients with liver histology characterized by fat accumulation and hepatic necroinflammation in the absence of excessive alcohol consumption [41]. Thus, the current terminology of “nonalcoholic fatty liver disease” is mainly derived from excluding alcohol-related heptopathology, focusing on what “does not” lead to this type of fatty liver rather than what “may lead to it.” This is mainly due to knowledge gaps in understanding the natural history of NAFLD, which also poses an obstacle to developing a clear approach to the care of patients with co-existing type 2 diabetes [42]. Furthermore, the inclusion of NAFLD among diabetes-related complications is a matter of debate, which is unlikely to be resolved without mechanistic studies that evaluate the relationship between both diseases. Moreover, a discussion about how to define NAFLD in the presence of a pre-existing type 2 diabetes is needed [43]. What is agreed on is that both conditions are related to obesity, subclinical inflammation, and insulin resistance, but the sequence of events is poorly identified.

#### Noninvasive assessment of NAFLD

The standards of care of the American Diabetes Association (ADA) recommend evaluating patients with type 2 diabetes or pre-diabetes, who have elevated alanine aminotransferase (ALT) or NAFL by ultrasonography (US) for NASH and liver fibrosis. The ADA guidelines suggest using vibration controlled transient elastography (VCTE) and noninvasive biomarkers for risk-stratification [44]. According to the American Association for the Study of Liver Diseases (AASLD), the diagnosis of NAFLD is defined by the presence of ≥5% hepatic steatosis either by imaging or histology with the absence of secondary causes of hepatic steatosis such as high alcohol consumption, monogenic hereditary disorders like Wilson’s disease or long-term use of steatogenic medications like methotrexate, amiodarone, and tamoxifen [2]. Liver biopsy remains the gold standard technique for diagnosing NASH and liver fibrosis; however, it is invasive, carries some intrinsic morbidity and mortality risk, may fail in staging the disease is subject to sampling error, and has reading variability [45].

In clinical practice, US is the recommended first-line imaging technique for diagnosing NAFLD; however, its sensitivity is reduced when hepatic fat content is < 20–33% [46, 47]. Other non-invasive tools have been developed for diagnosing NAFLD. These include magnetic resonance spectroscopy and magnetic resonance elastography. However, these tools are expensive, time-consuming, and are not considered cost-effective for large-scale NAFLD screening. In clinical research, recent data showed that magnetic resonance imaging–derived proton density fat fraction (MRI-PDFF) is a reliable, non-invasive alternative to conventional liver biopsy in assessing response to treatment in early-phase NASH trials [48]. On the other hand, VCTE is an imaging technology widely used at liver clinics as a simple aid for diagnosis and follow up of patients with NAFLD and other chronic liver diseases [49]. VCTE has the advantage of evaluating a portion of the liver that is 100-fold greater than that evaluated by needle biopsy and in much shorter time. The generic name for VCTE is Fibroscan® (Echosens Paris, France), which produces a quantifiable, reproducible liver stiffness measurement (LSM) expressed in kilopascals (kPa). A LSM value of > 9.8 kPa is consistent with the presence of advanced fibrosis/cirrhosis [50–52]. More recently, the growing interest in precision medicine led to the development of liquid biopsy tools. These are non-invasive, mechanism-based biomarkers that have the potential to eventually replace conventional needle biopsy for diagnosis, stage stratification, and monitoring of response to treatment in NASH and other chronic liver diseases [53]. Table 1 provides a summary of the most reliable and widely used imaging modalities for NAFLD diagnosis.

**Table 1:** Noninvasive imaging assessment of NAFLD and advanced fibrosis

Diagnostic modality	Advantages	Disadvantages
US^a^	• Noninvasive • Inexpensive • Widely available • Fair accuracy in moderate to severe hepatic steatosis (≥ S2)^a^	• ↓sensitivity when hepatic steatosis < 20–33%^a^ • Operator-dependent • ↓accuracy in patients with chronic liver disease or obesity
VCTE (CAP^a^ & LSM^b^)	• Noninvasive • Inexpensive • Widely available • Reproducible • Advanced fibrosis staging^b^	• Technical limitations in patients with ascites, morbid obesity, or ↑chest wall fat • Measurement failure
MRI-PDFF^a^ & MRE^b^	• Noninvasive • Quantification of hepatic steatosis^a^ (helpful in patients with ↓grade hepatic steatosis) • Excellent reproducibility • Advanced fibrosis staging^b^	• Expensive • Small sample volume/not convenient for patients with uneven fatty changes^a^

^a^steatosis assessment. ^b^ fibrosis assessment

*Abbreviations*: *US* Ultrasonography, *VCTE* Vibration-controlled transient elastography, *CAP* Controlled attenuation parameter, *LSM* liver Stiffness measurement, *MRI-PDFF* Magnetic resonance imaging-proton density fat fraction, *MRE* Magnetic resonance elastography

**Fig. 3:**
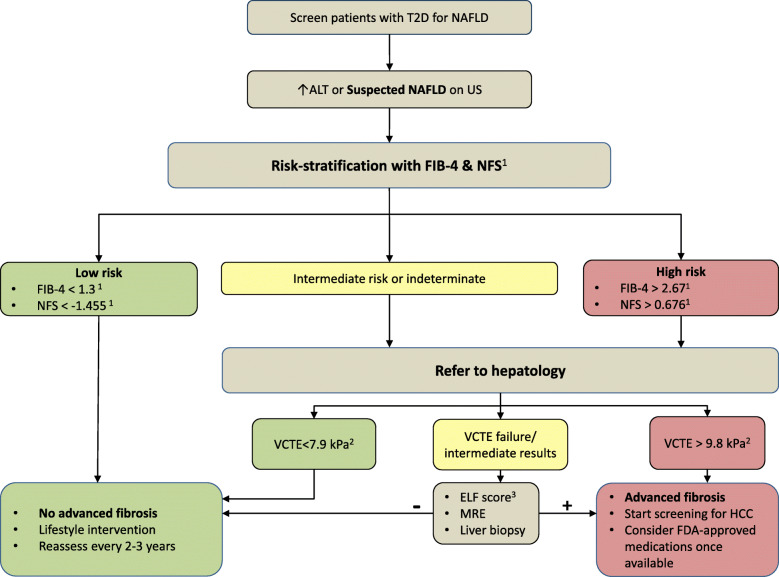
Proposed algorithm to screen patients with type 2 diabetes for NAFLD Patients with type 2 diabetes and suspected NAFLD can be risk-stratified using a combination of noninvasive scores/imaging. Indeterminate- and High-risk patients can then be prioritized for specialty referral for further investigation. ^1^Cut-off values reported by Angulo et al. [58]. NFS is calculated using the formula: NFS =  −1.675 + 0.037 – age (years) + 0.094 – BMI (kg/m^2^) + 1.13 × IFG/diabetes (yes = 1, no = 0) + 0.99 × AST/ALT ratio – 0.013 × platelet count (×10^9^/l) – 0.66 × albumin (g/dl). (https://nafldscore.com/). FIB-4 is calculated using the formula: FIB-4 = Age (years)×AST (U/L)/[PLT(109/L)×ALT^1/2^ (U/L)] (https://www.hepatitisc.uw.edu/page/clinical-calculators/fib-4). ^2^Cut-off values reported by Tapper et al. [52]. ^3^Rosenberg et al. [59]. Abbreviations: T2D, type 2 diabetes; NAFLD, nonalcoholic fatty liver disease; US, ultrasonography; ALT, alanine aminotransferase; FIB-4, fibrosis index-4; NFS, NAFLD fibrosis score; VCTE, vibration-controlled transient elastography; ELF, enhanced liver fibrosis; MRE, magnetic resonance elastography; HCC, hepatocellular carcinoma; FDA, US food and drug administration.

Many noninvasive scores that are simply calculated using routinely available labs and demographic data have been developed to predict the presence of suspected NAFLD [54, 55] including the hepatic steatosis index (HSI) [56], and fatty liver index (FLI) [57]. Other scores could predict the presence of advanced fibrosis (Table 2) such as the FIB-4 index [60], NAFLD fibrosis score (NFS) [61], the enhanced liver fibrosis (ELF) score [59] and alanine aspartate transferase (AST) to platelet ratio (APRI) [62]. Despite their poor sensitivity in detecting advanced fibrosis in patients with type 2 diabetes [63], these scores (FIB-4 is among best studied) [64, 65] have reasonable specificity and can be convenient for healthcare providers to assess patients with suspected NAFLD based on US or elevated levels of ALT [58, 66] (Fig. 3). It is important to recognize that patients with the NAFLD spectrum may still present with normal ALT levels including those with NASH, advanced fibrosis, and cirrhosis [67]. Normal ALT levels should therefore be taken with a grain of salt. One study proposed a stage-based approach that uses non-invasive scores alongside VCTE to risk-stratify patients with NAFLD and determine when to consider liver biopsy [68]. A more recent study by Davyduke et al. evaluated the impact of a “FIB-4 first” strategy to reduce the need for VCTE and hepatology referral [69]. Today, many investigational new drugs for NASH treatment are in phase III clinical trials, some of which might ultimately be approved by the U.S. Food and Drug Administration (FDA) as early as 2020.

**Table 2:** Demographic- and serum-based biomarkers for fibrosis staging

Biomarker	Components	Cut-offs to rule out/in advanced fibrosis
FIB-4 index [58]	Age, AST, ALT, and platelets	< 1.3 > 2.67
NAFLD fibrosis score [58]	Age, BMI, IFG and diabetes, AST-to-ALT ratio, platelets, and albumin	< - 1.455 > 0.676
Enhanced liver fibrosis test [59]	Age, hyaluronic acid, aminoterminal propeptide of type III collagen, and tissue inhibitor of matrix metalloproteinase 1	≥9.8

*Abbreviations*: *BMI* Body mass index, *IFG* Impaired fasting glucose, *AST* Aspartate aminotransferase, *ALT* Alanine aminotransferase, *FIB* Fibrosis index

NFS is calculated using the formula: NFS = − 1.675 + 0.037 – age (years) + 0.094 – BMI (kg/m^2^) + 1.13 × IFG/diabetes (yes = 1, no = 0) + 0.99 × AST/ALT ratio – 0.013 × platelet count (× 10^9^/l) – 0.66 × albumin (g/dl). (https://nafldscore.com/)

FIB-4 is calculated using the formula: FIB-4 = Age (years) × AST (U/L)/[PLT(10^9^/L) × ALT^1/2^ (U/L)] (https://www.hepatitisc.uw.edu/page/clinical-calculators/fib-4)

We strongly believe that increased awareness of NAFLD and improved disease recognition among diabetologists would help in identifying patients with prediabetes and diabetes who might benefit from risk factor modification or emerging novel therapies to slow the progression of CVD and hepatic complications. Using validated risk scores like FIB-4 [64, 65] within electronic health records, similar to eGFR calculation, maybe a good initial step. In Fig. 3, we suggest an algorithm to aid diabetologists and primary care providers in screening patients with type 2 diabetes for NAFLD and advanced fibrosis. The cost-effectiveness of screening for NAFLD may still be debated; however, we believe proactive screening is better than passive waiting for fibrosis progression.

## Therapeutic approaches

### Lifestyle intervention

Lifestyle intervention with diet, exercise, and behavioral modification is the initial step in managing type 2 diabetes [70]. This also applies to patients with NAFLD [71]. Steatosis can be reduced by as little as 3–5% weight loss. On the other hand, 7–9% weight loss is typically needed to reduce inflammation, while 10% is required to initiate fibrosis regression [72]. Although it is widely thought that sustainable weight loss through lifestyle modification is often difficult to achieve, a multidisciplinary approach to lifestyle intervention in patients with type 2 diabetes has been shown to induce weight loss that is both maintainable and clinically meaningful. We previously reported that 53% of participants in a real-world, multidisciplinary lifestyle intervention program who achieved an average of ≥7% weight loss at 1 year were able to maintain up to 9% weight loss at 5 years [73]. On the other hand, a Mediterranean eating pattern was shown to reduce hepatic steatosis and IR independent of weight loss in insulin-resistant individuals without diabetes but with biopsy-proven NAFLD [74]. Other strategies to induce weight loss, such as bariatric surgery and endoscopic bariatric procedures may be considered in NAFLD [2, 75]. In patients with type 2 diabetes, bariatric surgery was shown to reduce body weight, HbA1c, insulin resistance, and has led to partial or complete diabetes remission in some cases [76, 77]. More recently, duodenal mucosal resurfacing (DMR), a novel and minimally invasive endoscopic procedure, improved glycemic and hepatic indices in patients with type 2 diabetes, which shows promise of possible benefits in patients with NAFLD [78]. More data are still needed regarding the long-term efficacy of bariatric surgery and DMR on histologic severity and disease progression in patients with NASH [75, 79].

### Diabetes pharmacotherapy for NAFLD treatment

The dynamic association between NAFLD and hepatic IR has led to the experimentation of several diabetes medications for the treatment of NAFLD. These trials generated knowledge that may support future management paradigms for NAFLD in patients with diabetes, but further questions need to be answered.

#### Metformin

An adenosine monophosphate (AMP)-activated protein kinase (AMPK) activator; metformin is the first-line pharmacologic treatment for prediabetes and type 2 diabetes. Although several randomized controlled trials (RCTs) reported that metformin did not improve histologic features of NAFLD [80, 81], metformin may lower the risk of HCC in patients with diabetes [82]. In a recent translational study that included lung biopsies of humans with idiopathic pulmonary fibrosis (IPF) and a bleomycin mouse model (an experimental mouse model of lung fibrosis), AMPK activity was lower in fibrotic foci associated with active myofibroblasts. Moreover, the study reported that pharmacological activation of AMPK with metformin can reverse established fibrosis by facilitating deactivation and apoptosis of myofibroblasts [83]. In a more recent retrospective analysis of 191 patients with diabetes and biopsy-proven NASH and bridging fibrosis or compensated cirrhosis, metformin use was linked to lower risk of overall mortality and liver transplant (HR: 0·42; 95% CI: 0·24–0·74, *p* = 0·003) and HCC (sHR: 0·25; 95% CI: 0·11–0·58, *p* = 0·001) [84]. These findings may pave the way for studying AMPK activators in NAFLD, such as PXL770, which is being evaluated in a randomized clinical trial versus placebo to assess its effects on liver fat reduction after 12 weeks of treatment (ClinicalTrials.gov Identifier: NCT03763877). Metformin should be used with caution in patients with an estimated glomerular filtration rate (eGFR) < 45 mL/min, and possibly discontinued if eGFR drops < 30.

#### Glucagon-like Peptide-1 receptor agonists

The glucagon-like peptide-1 (GLP-1) receptor agonist liraglutide was studied in a recent multicenter, double-blind, randomized, placebo-controlled phase two study in patients with NASH (the LEAN study) [85]. NASH resolution was observed in nine patients (39%) in the liraglutide group in comparison to two patients (9%) in the placebo group (*p* = 0·019). Semaglutide, a longer-acting GLP-1 receptor agonist, is currently being studied in a larger sample size of 288 patients with NASH (ClinicalTrials.gov Identifier: NCT02970942). Semaglutide is also being studied in combination with other medications that inhibit hepatic DNL and affect bile acid-enterohepatic access (ClinicalTrials.gov Identifier: NCT03987074). There is conflicting evidence on risk of acute pancreatitis with the use of GLP-1 receptor agonists [86, 87]. Patients with type 2 diabetes and NASH should be made aware of these possible risks.

#### Thiazolidinediones

In a study that randomized 247 non-diabetic patients with NASH to either 30 mg of pioglitazone daily, 800 IU of vitamin E daily, or placebo, pioglitazone was not superior to placebo in improving histologic features of NASH after 96 weeks of intervention. However, the study showed that pioglitazone use was associated with significant improvements in hepatic steatosis, ALT, and AST compared to placebo [88]. In a more recent RCT that randomized 101 patients with prediabetes or type 2 diabetes and biopsy-proven NASH to receive either 45 mg of pioglitazone daily or placebo for 72 weeks, 51% of patients in the pioglitazone arm had resolution of NASH and improvement in several histologic features, including liver fibrosis [89]. Currently, Pioglitazone is the only diabetes medication included in recent guidance from the AASLD to treat patients with biopsy-proven NASH with or without type 2 diabetes [2, 90]; however, it should be prescribed with caution given its safety profile (potential weight gain, and risk of bladder cancer, bone loss, and congestive heart failure) [91].

### Sodium-glucose co-transporter 2 inhibitors

These medications inhibit glucose reabsorption in the proximal tubule, which leads to significant loss of glucose and calorie in the urine, resulting in improved insulin sensitivity, weight reduction, and potentially a reduction in liver fat content [92]. Previous reports linked remogliflozin to a 30–40% reduction in ALT levels in patients with abnormal baseline ALT [93]. canagliflozin [94] and dapagliflozin [95] also showed benefits in reducing serum aminotransferases. In a recent RCT that randomized 50 patients with type 2 diabetes and NAFLD to receive either standard diabetes care and empagliflozin or standard diabetes care only, liver fat, as measured by MRI-PDFF, decreased significantly in the empagliflozin arm compared to the comparative arm [96]. Further RCTs are needed to determine the effect of SGLT2 inhibitors on liver histology in NASH.

#### Treatment considerations

There is evidence, although not conclusive, that the use of insulin and oral insulin secretagogues, including sulfonylureas, may be associated with an increased risk of HCC in patients with type 2 diabetes [97, 98] possibly through insulin-mediated cancer cell proliferation [99]. Therefore, similar to the recent recommendation that CVD risk reduction should be taken into account when treating patients with type 2 diabetes, It is important to take into consideration the risk of NAFLD progression to HCC. When primary care providers see patients with type 2 diabetes and NAFLD being treated with metformin and glipizide, only it may be plausible to replace glipizide with a GLP-1 or SGLT-2 agent. This is important to consider, but caution is advised.

Finally, combination therapy is projected to be the future of NASH management, particularly in patients with co-existing type 2 diabetes. One attracting combination to improve NASH while reducing CVD risk may include low-dose pioglitazone with either a GLP-1 analogue or SGLT-2 inhibitor; however, future studies are needed to explore the efficacy of such combination [90].

### Tipping the scale-novel NASH therapies in the pipeline

The quest for a NASH-specific treatment has been harnessing significant attention from federal and private funders as well as the pharmaceutical industry. As of August 15, 2019, there were 750 NAFLD trials registered on clinicaltrilas.gov. Earlier in 2019, results from the STELLAR-3, a phase three, randomized, double-blind, placebo-controlled study which evaluated the safety and efficacy of selonsertib, an apoptosis signal-regulating kinase 1 (ASK1) inhibitor showed no superiority to placebo in the primary endpoint of a ≥ 1-stage histologic improvement in fibrosis without worsening of NASH in patients with bridging NASH-fibrosis (F3)(ClinicalTrials.gov Identifier: NCT03053050).

The farnesoid X receptor (FXR) agonist obeticholic acid (OCA) is the most advanced drug in the pipeline. FXR is a nuclear receptor with high expression in the liver and small intestine [100]. FXR naturally binds to bile acids [100], and they jointly regulate lipid/ glucose homeostasis, promote insulin sensitivity, and potentially modify liver fibrosis [101].

In phase two and three trials in patients with NASH and advanced fibrosis, OCA showed efficacy on fibrosis regression, paving the way for potential FDA approval by 2020 [102, 103]. In the phase 3 REGENERATE trial (NCT02548351), that randomized patients to receive OCA at 10 mg or 25 mg daily or placebo, the interim analysis at 18 months revealed significant improvement in fibrosis by one stage in patients on OCA 25 mg daily compared to those in the placebo arm (23·1% vs 11·9% *P* = 0·0002) [103]. The fact that fibrosis improvement by one stage occurred in less than one-quarter of patients provides a strong rationale for the need for combination therapy with other drugs to increase efficacy.

Multiple investigational new drugs show promising potential in modifying NASH and fibrosis progression and are now in phase 3 clinical trials (Table 3). A dual peroxisome proliferator-activated receptor (PPAR) α/δ agonist, elfibranor regulates glucose homeostasis and lipid metabolism and reduces inflammation, potentially modifying fibrosis [104] (NCT02704403). Resmetirom, a liver-directed thyroid hormone receptor-β agonist, increases hepatic fat metabolism and reduces lipotoxicity, potentially improving NASH [105] (NCT03900429). Another agent in clinical trials is aramchol, a synthetic fatty-acid/bile-acid conjugate, which reduces hepatic fat through downregulation of stearoyl-CoA desaturase-1, a fatty acid synthetic enzyme in hepatocytes. The anti-fibrotic effect of aramchol stems from its upregulation of PPAR δ in hepatic stellate cells (HSCs), the primary fibrogenic cell type in the liver [106] (NCT04104321). Lastly, cenicriviroc, a dual c-c chemokine receptor 2/5 antagonist which blocks intrahepatic macrophage trafficking and may confer anti-fibrotic effects through de-activation of HSCc [107, 108] (NCT03028740). These agents are currently being studied. If any deemed effective, an expected FDA-approval in late 2020 would be looming. Future studies should explore the efficacy of combined anti-fibrotic and anti-diabetic therapy in the management of patients with type 2 diabetes and NASH/fibrosis [90].

**Table 3:** NASH therapies in clinical trials

	Description	Target	Phase	Duration
Obeticholic acid	Semisynthetic bile acid	FXR agonist	III^a^	72 weeks
Elafibranor	Small-molecule	Dual PPAR α/δ agonist	III	72 weeks
Resmetirom	Small-molecule	THR β agonist	III	52 weeks
Aramchol	Synthetic FABAC	SCD-1 modulator	III	52 weeks
Cenicriviroc	Small-molecule	Dual CCR2/CCR5 antagonist	III	48 weeks

*Abbreviations*: *NASH* Nonalcoholic steatohepatitis, *FXR* Farnesoid x receptor, *PPAR* Peroxisome proliferator-activated receptor, *THR* Thyroid hormone receptor, *FABAC* Fatty-acid/bile-acid conjugate, *SCD* Stearoyl-CoA desaturase, *CCR* C-c chemokine receptor

^a^Topline results demonstrated significant improvement in fibrosis by 1 stage in patients on OCA 25 mg daily compared to those in the placebo arm [103]

## Conclusions

The prevalence of NASH among patients with type 2 diabetes is high, putting them at a significantly higher risk for developing end-stage liver disease, HCC, and CVD. Increased awareness about NASH and NASH-related complications is warranted among diabetologists, especially with the prospective induction of NASH-specific therapies. We believe an interdisciplinary approach is needed for the care of patients with type 2 diabetes and NAFLD starting with early identification through noninvasive biomarkers and imaging modalities in the diabetes clinic to lifestyle modification and NASH-specific therapy in the hepatology clinic.

## Data Availability

Data sharing is not applicable to this article as no data sets were generated or analyzed during the current study.

## Abbreviations

NAFLD: Nonalcoholic fatty liver disease
NAFL: Nonalcoholic fatty liver
NASH: Nonalcoholic steatohepatitis
HCC: Hepatocellular carcinoma
CVD: Cardiovascular disease
IR: Insulin resistance
FFAs: Free fatty acids
DNL: De novo lipogenesis
MetS: Metabolic syndrome
US: Ultrasonography
VCTE: Vibration controlled transient elastography
AASLD: American Association for the Study of Liver Diseases
MRI-PDFF: Magnetic resonance imaging–derived proton density fat fraction
LSM: Liver stiffness measurement
HSI: Hepatic steatosis index
FLI: Fatty liver index
NFS: NAFLD fibrosis score
AST: Aspartate aminotransferase
APRI: AST to platelet ratio
ALT: Alanine aminotransferase
DMR: Duodenal mucosal resurfacing
AMP: Adenosine monophosphate
AMPK: Activated protein kinase
RCTs: Randomized controlled trials
IPF: Idiopathic pulmonary fibrosis
GLP-1: Glucagon-like peptide-1
ASK1: Apoptosis signal-regulating kinase 1
FXR: Farnesoid X receptor
OCA: Obeticholic acid
PPAR: Peroxisome proliferator-activated receptor
HSCs: Hepatic stellate cells
